# 继发急性髓系白血病异基因造血干细胞移植疗效及预后影响因素分析

**DOI:** 10.3760/cma.j.cn121090-20230929-00151

**Published:** 2024-01

**Authors:** 晓琳 袁, 一波 吴, 晓露 宋, 怡 陈, 滢 陆, 晓瑜 来, 继敏 施, 丽珍 刘, 妍敏 赵, 建 余, 露欣 杨, 建平 蓝, 真 蔡, 河 黄, 依 罗

**Affiliations:** 1 浙江大学医学院附属第一医院骨髓移植中心、良渚实验室、浙江大学血液学研究所、浙江省干细胞与细胞免疫治疗工程实验室，杭州 310003 Bone Marrow Transplantation Center, the First Affiliated Hospital, Zhejiang University School of Medicine; Liangzhu Laboratory; Institute of Hematology, Zhejiang University; Zhejiang Province Engineering Laboratory for Stem Cell and Immunity Therapy, Hangzhou 310003, China; 2 浙江省人民医院，杭州 310014 Zhejiang Provincial People's Hospital, Hangzhou 310014, China; 3 温州医科大学附属第一医院，温州 325035 The First Affiliated Hospital of Wenzhou Medical University, Wenzhou 325035, China; 4 宁波大学附属人民医院，宁波 315000 People's Hospital Affiliated to Ningbo University, Ningbo 315000, China

**Keywords:** 白血病，髓系，急性, 继发白血病, 造血干细胞移植, 生存, 复发, Leukemia, myeloid, acute, Secondary leukemia, Hematopoietic stem cell transplantation, Survival, Relapse

## Abstract

**目的:**

探讨成人继发急性髓系白血病（AML）接受异基因造血干细胞移植（allo-HSCT）的疗效及预后影响因素。

**方法:**

多中心回顾性临床研究。纳入2014年1月至2022年11月因继发AML在浙江省造血干细胞移植协作组4个中心接受allo-HSCT的18岁以上的成人患者，并进行疗效及预后影响因素分析。

**结果:**

共纳入95例患者，其中66例（69.5％）为骨髓增生异常综合征（MDS）转化AML（MDS-AML），4例（4.2％）为MDS/骨髓增殖性肿瘤（MPN）转化AML（MDS/MPN-AML），25例（26.3％）为治疗相关AML（tAML）。所有患者的3年累积复发率（CIR）、无白血病生存（LFS）率和总生存（OS）率分别为18.6％（95％*CI* 10.2％～27.0％）、70.6％（95％*CI* 60.8％～80.4％）和73.3％（95％*CI* 63.9％～82.7％）。M-AML（包括MDS-AML、MDS/MPN-AML）组和tAML组的3年CIR分别为20.0％和16.4％（*P*＝0.430）；3年LFS率分别为68.3％和75.4％（*P*＝0.176）；3年OS率分别为69.7％和75.4％（*P*＝0.233），两组间差异均无统计学意义。TP53突变组和无TP53突变组的3年CIR分别为60.0％和13.7％（*P*＝0.003），3年LFS率分别为20.0％和76.5％（*P*＝0.002），3年OS率分别为40.0％和77.6％（*P*＝0.002）。根据2022欧洲白血病网（ELN2022）危险分层，低危、中危和高危3组患者的3年CIR分别为8.3％、17.8％和22.6％（*P*＝0.639），3年LFS率分别为91.7％、69.5％和65.6％（*P*＝0.268），3年OS率分别为91.7％、71.4％和70.1％（*P*＝0.314）。多因素分析表明，移植时疾病未缓解和伴有TP53突变是影响患者CIR、LFS和OS的独立危险因素。

**结论:**

M-AML组（MDS-AML、MDS/MPN-AML）与tAML组患者allo-HSCT预后相近。移植时疾病未缓解和伴有TP53突变是不良预后因素。ELN2022危险分层对继发AML患者allo-HSCT预后的预测价值有限。

## Abstract (en)

**Objective:**

To evaluate the efficacy and prognostic factors of allogeneic hematopoietic stem cell transplantation（allo-HSCT）in patients with secondary acute myeloid leukemia（sAML）.

**Methods:**

In this multicenter, retrospective clinical study, adult patients aged ≥18 years who underwent allo-HSCT for sAML at four centers of the Zhejiang Hematopoietic Stem Cell Transplantation Collaborative Group from January 2014 to November 2022 were included, and the efficacy and prognostic factors of allo-HSCT were analyzed.

**Results:**

A total of 95 patients were enrolled; 66（69.5%）had myelodysplastic syndrome-acute myeloid leukemia（MDS-AML）, 4（4.2%）had MDS/MPN-AML, and 25（26.3%）had therapy-related AML（tAML）. The 3-year CIR, LFS, and overall survival（OS）rates were 18.6%（95% *CI* 10.2%−27.0%）, 70.6%（95% *CI* 60.8%−80.4%）, and 73.3%（95% *CI* 63.9%−82.7%）, respectively. The 3-year CIRs of the M-AML group（including MDS-AML and MDS/MPN-AML）and the tAML group were 20.0% and 16.4%, respectively（*P*＝0.430）. The 3-year LFSs were 68.3% and 75.4%, respectively（*P*＝0.176）. The 3-year OS rates were 69.7% and 75.4%, respectively（*P*＝0.233）. The 3-year CIRs of the groups with and without TP53 mutations were 60.0% and 13.7%, respectively（*P*＝0.003）; the 3-year LFSs were 20.0% and 76.5%, respectively（*P*＝0.002）; and the 3-year OS rates were 40.0% and 77.6%, respectively（*P*＝0.002）. According to European LeukmiaNet 2022（ELN2022）risk stratification, the 3-year CIRs of patients in the low-, intermediate-, and high-risk groups were 8.3%, 17.8%, and 22.6%, respectively（*P*＝0.639）. The three-year LFSs were 91.7%, 69.5%, and 65.6%, respectively（*P*＝0.268）. The 3-year OS rates were 91.7%, 71.4%, and 70.1%, respectively（*P*＝0.314）. Multivariate analysis revealed that advanced disease at allo-HSCT and TP53 mutations were independent risk factors for CIR, LFS, and OS.

**Conclusion:**

There was no significant difference in the prognosis of patients who underwent allo-HSCT among the MDS-AML, MDS/MPN-AML, and tAML groups. Advanced disease at transplantation and TP53 mutations were poor prognostic factors. ELN2022 risk stratification had limited value for predicting the prognosis of patients with sAML following allo-HSCT.

继发急性髓系白血病（sAML）是一组由多种疾病亚型组成的一组异质性疾病，一般包括治疗相关AML（tAML）和既往血液系统疾病转化而来的AML，约占所有AML中的25％～30％[1]–[2]。最近WHO和国际共识分类（ICC）分别更新了AML的分类[3]–[4]。WHO分类定义的sAML包括细胞毒性药物治疗后的AML和胚系易感性相关的AML，而骨髓增生异常综合征（MDS）和MDS/骨髓增殖性肿瘤（MPN）转化的AML则归类为MDS相关AML（AML-MR）。ICC共识已经删除了“继发急性髓系白血病”的命名，将MDS转化、MDS/MPN转化及tAML作为诊断限定词。本研究纳入MDS-AML、MDS/MPN-AML和tAML，为了便于描述，本研究将这些疾病归为sAML。有研究显示，与新诊断的AML相比，sAML通常与更差的预后相关[1],[5]，sAML预后不佳多是由于高危疾病特征如疾病未缓解、不良遗传学、分子学特征、可检测残留病（MRD）阳性等[1],[6]–[7]。异基因造血干细胞移植（allo-HSCT）为这部分患者提供了治愈的可能和途径。然而目前移植治疗sAML的预后并不清楚[7]–[9]，本研究通过对多中心接受allo-HSCT 治疗的sAML患者进行回顾性分析，描述sAML患者特征，探讨sAML患者移植预后及影响因素。

## 病例与方法

一、病例资料

回顾性收集2014年1月至2022年11月在浙江大学医学院附属第一医院、浙江省人民医院、温州医科大学附属第一医院、宁波大学附属人民医院接受首次allo-HSCT且≥18岁的MDS-AML、MDS/MPN-AML和tAML的连续病例。MDS-AML、MDS/MPN及tAML的诊断参考2022 ICC[4]。在原发病的放化疗后与诊断AML之间发生MDS或MDS/MPN的患者被归为tAML。本研究经浙江大学医学院附属第一医院科研伦理审查委员会审批通过（批件号：2021-IIT-690），所有患者均签署知情同意书，所有患者末次随访时间为2023年8月31日，通过查阅患者住院病历、门诊随访记录和电话随访获得患者生存资料。

二、移植流程

73例患者接受单倍型allo-HSCT，13例接受亲缘全相合allo-HSCT，余9例接受非亲缘allo-HSCT。1例接受骨髓来源allo-HSCT，2例接受骨髓联合外周血来源allo-HSCT，余92例均接受外周血来源allo-HSCT。86例患者接受以白消安（Bu）+环磷酰胺（Cy）±阿糖胞苷（Ara-C）为基础的清髓性预处理方案，9例患者接受以氟达拉滨（Flu）+ Bu为基础的减剂量预处理方案。所有患者移植后均接受环孢素A、霉酚酸酯以及短程甲氨蝶呤预防移植物抗宿主病（GVHD）。

三、定义和评估

疾病危险分层采用ELN2022危险分层标准分为低危、中危和高危[10]。完全缓解（CR）定义为骨髓原始细胞<5％，外周血没有出现原始细胞，且无髓外病灶。复发定义为骨髓原始细胞≥5％、外周血中出现原始细胞或出现髓外浸润[10]。非复发死亡定义为因非复发或非疾病进展原因造成的死亡。对骨髓标本采用流式细胞术（FCM）检测白血病相关免疫表型（LAIP）和聚合酶链反应（PCR）检测白血病相关基因监测MRD。MRD阳性定义基于ELN共识[11]。未缓解患者不纳入MRD评估。

四、研究终点及统计学处理

主要观察终点是累积复发率（CIR）、总生存（OS）率和无白血病生存（LFS）率。OS期定义为从移植当天开始至末次随访或死亡的时间。LFS期定义为从移植当天开始至末次随访、白血病复发或者死亡的时间。使用竞争风险模型方法分析CIR，将非复发死亡作为竞争事件。OS和LFS采用 Kaplan-Meier方法计算，生存曲线比较采用Log-rank检验。计量资料比较采用Mann-Whitney *U*检验，计数资料比较采用*χ*^2^检验或Fisher精确检验。将疾病类型、年龄和单因素分析中差异具有统计学意义的因素纳入多因素分析。采用Cox回归模型进行多因素分析。使用SPSS 22.0及R（version 4.2.1）软件进行统计分析。*P*<0.05为差异有统计学意义。

## 结果

一、患者基本特征

本研究共纳入95例连续病例，其中66例（69.5％）为MDS-AML，4例（4.2％）为MDS/MPN-AML，25例（26.3％）为tAML。移植时中位年龄为47（IQR：32～55）岁。存活患者移植后中位随访时间为1 198（IQR：649～1 988）d。按照疾病类型，将患者分为M-AML组（MDS-AML和MDS/MPN-AML）和tAML组。MDS/MPN-AML患者原发病均为慢性粒-单核细胞白血病（CMML），tAML患者原发病最多的为乳腺癌（8例），其次为淋巴瘤（5例）和结直肠癌（4例）。M-AML组患者中位年龄为46（IQR：32～55）岁，tAML组患者中位年龄为50（IQR：35～56）岁。tAML组诊断时WBC、骨髓原始细胞比例、造血干细胞移植并发症指数（HCT-CI）评分较M-AML组更高。两组患者移植时年龄、诊断时HGB、PLT、移植前血清铁蛋白、供者类型、预处理方案差异均无统计学意义（表1）。

**表1:** 95例接受异基因造血干细胞移植的继发急性髓系白血病（sAML）患者临床特征

临床特征	M-AML（70例）	tAML (25例）	统计量	*P*值
年龄[岁，*M*（IQR）]	46（32~55）	50（35~56）	−0.892	0.372
女性[例(%)]	33（47.1）	20（80.0）	8.063	0.005
诊断时WBC [×10^9^/L，*M*（IQR）]	2.9（1.8~7.1）	6.7（2.7~31.4）	−2.596	0.009
诊断时HGB [g/L，*M*（IQR）]	75（63~97）	90（64~119）	−1.360	0.174
诊断时PLT[×10^9^/L，*M*（IQR）]	67（28~109）	65（23~128）	−0.164	0.870
骨髓原始细胞[%，*M*（IQR）]	34.0（24.0~50.0）	51.0（32.5~76.7）	−2.646	0.008
移植前铁蛋白[ng/ml，*M*（IQR）]	1 097.1（390.9~2 000.0）	697.5（352.6~1 836.8）	−0.674	0.501
HCT-CI≥1[例(%)]	18（25.7）	23（92.0）	32.993	<0.001
ECOG评分[例(%)]			4.136	0.042
0~1	40（57.1）	20（80.0）		
2~4	30（42.9）	5（20.0）		
ELN2022危险分层[例(%)]			15.273	<0.001
低危	4（5.7）	8（32.0）		
中危	42（60.0）	6（24.0）		
高危	24（34.3）	11（44.0）		
移植时疾病状态[例(%)]			9.391	0.009
CR_1_	31（44.3）	17（68.0）		
≥CR_2_	10（14.3）	6（24.0）		
nCR	29（41.4）	2（8.0）		
供者类型[例(%)]			0.465	0.793
亲缘全相合	9（12.9）	4（16.0）		
单倍体	55（78.6）	18（72.0）		
非亲缘	6（8.6）	3（12.0）		
清髓性预处理[例(%)]	64（91.4）	22（88.0）	Fisher	0.694
移植前MRD[例(%)]			11.202	0.004
MRD阳性	13（18.6）	4（16.0）		
MRD阴性	28（40.0）	19（76.0）		
nCR	29（41.4）	2（8.0）		
Post-MRD1阳性[例(%)]	12（17.4）	3（12.0）	Fisher	0.752
基因突变[例(%)]				
TET2	8（11.4）	7（28.0）	Fisher	0.062
NRAS	9（12.9）	3（12.0）	Fisher	1.000
ASXL1	9（12.9）	2（8.0）	Fisher	0.722
TP53	4（5.7）	6（24.0）	Fisher	0.019
FLT3-ITD	6（8.6）	3（12.0）	Fisher	0.694
DNMT3A	5（7.1）	3（12.0）	Fisher	0.430
RUNX1	6（8.6）	1（4.0）	Fisher	0.671
NPM1	3（4.3）	4（16.0）	Fisher	0.075
IDH2	5（7.1）	2（8.0）	Fisher	1.000

注 M-AML：骨髓增生异常综合征（MDS）转化的AML和MDS/骨髓增殖性肿瘤（MPN）转化的AML；tAML：治疗相关AML；IQR：四分位距；HCT-CI：造血干细胞移植并发症指数；ELN：欧洲白血病网；CR_1_：第1次完全缓解；CR_2_：第2次完全缓解；nCR：未完全缓解；ECOG：美国东部肿瘤协作组；MRD：可检测残留病；Post-MRD1：移植后1个月MRD

二、根据原发病分类的sAML患者的结局

所有患者总体的3年CIR、LFS率和OS率分别为18.6％（95％*CI* 10.2％～27.0％）、70.6％（95％*CI* 60.8％～80.4％）和73.3％（95％*CI* 63.9％～82.7％）。共有17例患者复发，其中M-AML组14例，中位复发时间为129（45～755）d；tAML组3例，中位复发时间为100（62～958）d。M-AML组和tAML组的3年CIR分别为20.0％（95％*CI* 10.0％～30.0％）和16.4％（95％*CI* 0～35.6％）（*P*＝0.430，图1A）；3年LFS率分别为68.3％（95％*CI* 56.9％～79.7％）和75.4％（95％*CI* 55.0％～95.8％）（*P*＝0.176，图1B）；3年OS率分别为69.7％（95％*CI* 58.3％～81.8％）和75.4％（95％*CI* 55.0％～95.8％）（*P*＝0.233，图1C），差异均无统计学意义。

**图1:**
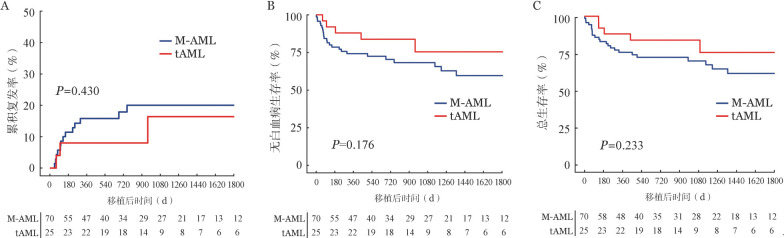
根据疾病类型分类的两组患者异基因造血干细胞移植后累积复发（A）、无白血病生存（B）和总生存（C）曲线 注 M-AML：骨髓增生异常综合征（MDS）转化的AML和MDS/骨髓增殖性肿瘤（MPN）转化的AML；tAML：治疗相关AML

三、根据基因突变分类的sAML患者的结局

95例患者中，最常伴有的突变依次为TET2 15例（15.8％）、NRAS 12例（12.6％）、ASXL1 11例（11.6％）、TP53 10例（10.5％）、FLT3-ITD 9例（9.5％）、DNMT3A 8例（8.4％）、RUNX1 7例（7.4％）、NPM1 7例（7.4％）和IDH2 7例（7.4％）（表1）。单因素分析发现，伴或不伴TP53突变的两组患者的CIR、LFS和OS差异均有统计学意义（*P*值均<0.05）。未发现TET2、NRAS、ASXL1、FLT3-ITD、DNMT3A、RUNX1、NPM1突变与患者CIR、LFS和OS相关（*P*值均>0.05）。伴有TP53突变的10例患者均接受亲缘单倍型造血干细胞移植。其中5例患者复发，中位复发时间为153（62～958）d，2例患者发生非复发死亡，均死于感染。TP53突变组和无TP53突变组的3年CIR分别为60.0％（95％ *CI* 13.5％～100％）和13.7％（95％ *CI* 13.1％～14.3％）（*P*＝0.003，图2A），3年LFS率分别为20.0％（95％ *CI* 0～51.4％）和76.5％（95％ *CI* 67.1％～85.9％）（*P*＝0.002，图2B），3年OS率分别为40.0％（95％ *CI* 10.6％～69.4％）和77.6％（95％ *CI* 68.2％～87.0％）（*P*＝0.002，图2C）。

**图2:**
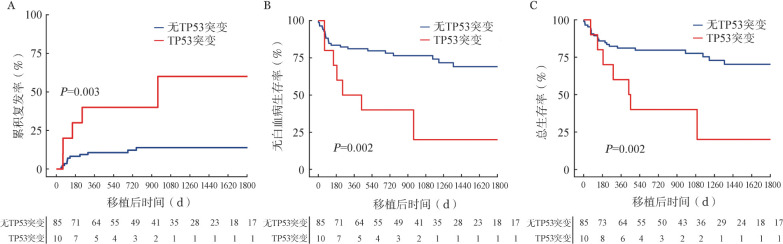
根据是否伴有TP53突变分类的两组患者异基因造血干细胞移植后累积复发（A）、无白血病生存（B）和总生存（C）曲线

四、根据ELN2022风险分层分类的sAML患者的结局

根据ELN2022危险分层，12例（12.6％）患者为低危，48例（50.5％）患者为中危，35例（36.8％）患者为高危。三组患者的3年CIR分别为8.3％（95％ *CI* 0～24％）、17.8％（95％ *CI* 6.3％～29.3％）和22.6％（95％ *CI* 6.6％～38.6％）（*P*＝0.639，图3A）。3年LFS率分别为91.7％（95％ *CI* 76.0％～100％）、69.5％（95％ *CI* 56.0％～83.0％）和65.6％（95％ *CI* 48.2％～83.0％）（*P*＝0.268，图3B）。3年OS率分别为91.7％（95％ *CI* 76.0％～100％）、71.4％（95％*CI* 57.9％～84.9％）和70.1％（95％ *CI* 54.4％～85.8％）（*P*＝0. 314，图3C）。三组患者CIR、LFS和OS差异无统计学意义。

**图3:**
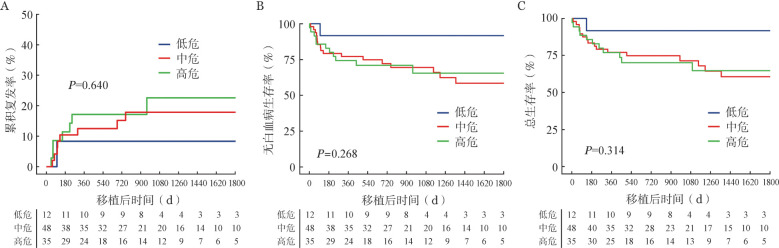
根据ELN2022风险分层分类的三组患者异基因造血干细胞移植后累积复发（A）、无白血病生存（B）和总生存（C）曲线

五、预后因素分析

单因素分析纳入的因素包括疾病类型（tAML对 M-AML）、年龄（以50岁为界）、性别、诊断时WBC（以10×10^9^/L为界）、骨髓原始细胞比例（以40％为界）、移植前铁蛋白（以1 000 ng/ml为界）、HCT-CI（≥1对0）、ELN2022危险分层、移植时疾病状态、供者类型、ECOG评分（≥2分对<2分）、预处理方案（减剂量对清髓性）、移植前MRD、移植后1个月MRD（Post-MRD1），以及TET2、NRAS、ASXL1、TP53、FLT3-ITD、DNMT3A、RUNX1、IDH2突变。结果显示移植前疾病未缓解、Post-MRD1阳性和TP53突变是CIR的影响因素。移植前疾病未缓解、美国东部肿瘤协作组（ECOG）评分≥2分、TP53突变与较差的LFS和OS显著相关（*P*值均<0.05）。

将疾病类型、年龄及以上*P*<0.05的因素纳入多因素分析，结果显示：移植前未缓解［CIR：*HR*＝10.66（95％*CI* 2.43～46.70），*P*＝0.002；LFS：*HR*＝13.75（95％*CI* 4.70～40.21），*P*<0.001；OS：*HR*＝8.04（95％*CI* 3.18～20.35），*P*＝0.004］和TP53突变［CIR：*HR*＝7.71（95％*CI* 2.43～24.49），*P*<0.001；LFS：*HR*＝5.05（95％*CI* 1.88～13.58），*P*＝0.001；OS：*HR*＝3.86（95％*CI* 1.60～9.33），*P*＝0.003］是影响CIR、LFS和OS的独立危险因素（表2）。

**表2:** 影响接受异基因造血干细胞移植的继发急性髓系白血病（sAML）患者CIR、LFS和OS的多因素分析

影响因素	CIR		LFS		OS	
	*HR*（95% *CI*）	*P*值	*HR*（95% *CI*）	*P*值	*HR*（95% *CI*）	*P*值
疾病类型（tAML对M-AML）	0.73（0.17~3.19）	0.671	0.67（0.19~2.38）	0.531	1.01（0.30~3.42）	0.987
年龄≥50岁	1.96（0.61~6.32）	0.261	2.13（0.95~4.75）	0.066	1.50（0.67~3.39）	0.327
ECOG评分≥2分	0.57（0.18~1.81）	0.341	2.12（0.78~5.77）	0.142	2.01（0.75~5.41）	0.163
Post-MRD1阳性	2.43（0.88~6.75）	0.087				
移植时疾病状态						
≥CR对CR_1_	1.36（0.16~7.44）	0.782	2.14（0.57~8.03）	0.259	1.45（0.36~5.83）	0.857
nCR对CR_1_	10.66（2.43~46.70）	0.002	13.75（4.70~40.21）	<0.001	8.04（3.18~20.35）	0.004
TP53突变	7.71（2.43~24.49）	<0.001	5.05（1.88~13.58）	0.001	3.86（1.60~9.33）	0.003

注 CIR：累积复发率；LFS：无白血病生存；OS：总生存；tAML：治疗相关AML；M-AML：骨髓增生异常综合征（MDS）转化的AML和MDS/骨髓增殖性肿瘤（MPN）转化的AML；nCR：未完全缓解；ECOG：美国东部肿瘤协作组；Post-MRD1：移植后1个月微小残留病

## 讨论

本研究通过多中心回顾性数据发现sAML患者接受allo-HSCT治疗可以获得较好的长期生存，M-AML与tAML两组患者结局差异无统计学意义。tAML组患者女性占80％，这与女性更容易患乳腺癌及妇科肿瘤相关。与M-AML相比，tAML组患者HCT-CI评分及诊断AML时原始细胞比例更高。但未发现HCT-CI评分及诊断时原始细胞比例对结局的影响。M-AML组移植时ECOG评分更低，单因素分析发现ECOG评分与LFS和OS相关，但多因素分析发现EOCG评分不是LFS和OS的独立危险因素。本研究未发现年龄与移植结局的相关性，所有患者的中位年龄47（18～68）岁，比既往研究报道的sAML年龄略小[12]–[13]，这可能与较多高龄患者不适合接受allo-HSCT有关。

Nagler等[13]的研究显示，第1次完全缓解（CR_1_）时接受移植的sAML患者3年CIR和LFS率分别为35％和41.6％。原发诱导失败、移植时未缓解的患者3年CIR分别为45.8％和46.8％，3年LFS率分别为29.8％和31.8％。EBMT急性白血病工作组的大样本研究结果显示，sAML移植后2年CIR为31.4％，2年LFS率为49.7％。Nilsson等[14]基于人群的一项队列研究报道显示，接受移植的 tAML和MDS-AML患者 3年OS率分别为42％和31％。本组患者移植后3年CIR和LFS率分别为18.6％和70.6％，患者移植预后优于既往研究，这可能与本研究纳入的患者相对较年轻，且有77％的患者接受了单倍型造血干细胞移植，可能发挥了相对较强的移植物抗白血病效应等相关。Nagler等[12]最近的研究也表明，CR_1_时接受移植后环磷酰胺（PTCy）模式下的单倍型造血干细胞移植可能能够克服sAML的不良预后，2年CIR和LFS率分别为20.6％和58.4％，与新诊断AML移植后结局差异无统计学意义。

本研究发现伴有TP53突变是移植后CIR、LFS和OS的独立危险因素。即便接受了allo-HSCT，预后也极差，目前ICC也已将具有TP53突变的AML作为一个单独分类[4]。目前亟需一种能够有效改善伴TP53突变的AML预后的新疗法。最近Othman等[15]的研究结果显示，治疗相关的和新诊断的伴有NPM1突变的AML具有重叠的特征和相似的预后。本研究中有7例患者携带NPM1突变，截至末次随访日期，7例患者均为无复发生存状态。与PETHEMA研究结果一致[1]，本研究也未发现FLT3-ITD对预后的影响。MRD是AML患者的一个重要预后指标。EBMT一项研究纳入318例18～75岁CR_1_时接受allo-HSCT的成人sAML患者，发现移植前MRD状态对移植后结局没有显著影响[16]。Rodríguez-Arbolí等[17]的一项研究发现，sAML患者移植前MRD阳性与较高的复发风险和较低的生存率相关。本研究未发现移植前MRD与结局的相关性，但是我们发现Post-MRD1阳性患者复发风险较高（*HR*＝3.8，*P*<0.05）。

目前尚无研究评估ELN2022风险分层是否可以预测继发AML移植后结局。本研究发现，ELN2022低危、中危和高危三组患者3年CIR、LFS和OS差异无统计学意义，ELN2022并不能很好地预测sAML患者移植预后。这可能与ELN危险分层是基于诊断时遗传学和分子学特征并不能代表移植时疾病危险程度相关，还需要结合其他指标如移植时疾病状态等来指导预后分层；也可能是由于样本量较小尚没有得出统计学差异，未来还需要大样本研究来验证。

本研究也存在一些局限性：首先本研究为回顾性研究，可能存在选择性偏倚，但我们选择了多中心连续病例，试图减少选择性偏倚。由于sAML患者病史及治疗方案较复杂，以及可及性的限制，本研究未对移植前既往治疗情况进行分析。

综上所述，sAML患者接受allo-HSCT治疗可以获得较好的长期生存，本研究结果显示M-AML与tAML移植预后相似，移植时疾病未缓解和伴有TP53突变是移植预后的独立危险因素。ELN2022并不能预测移植预后，还需要结合疾病状态等其他因素来指导预后分层。影响sAML移植预后的因素未来还需要多中心前瞻性研究来证实。
